# A genetic variant in *osteoprotegerin* is associated with progression of joint destruction in rheumatoid arthritis

**DOI:** 10.1186/ar4558

**Published:** 2014-05-07

**Authors:** Rachel Knevel, Diederik PC de Rooy, Tore Saxne, Elisabet Lindqvist, Martha K Leijsma, Nina A Daha, Bobby PC Koeleman, Roula Tsonaka, Jeanine J Houwing-Duistermaat, Joris JM Schonkeren, Rene EM Toes, Tom WJ Huizinga, Elisabeth Brouwer, Anthony G Wilson, Annette HM van der Helm-van Mil

**Affiliations:** 1 Department of Rheumatology, Leiden University Medical Center, Leiden, the Netherlands; 2 Department of Rheumatology, Lund University, Lund, Sweden; 3 Department of Rheumatology and Clinical Immunology, University of Groningen, University Medical Center Groningen, Groningen, the Netherlands; 4 Complex Genetics Section, Department of Medical Genetics, University Medical Centre Utrecht, Utrecht, the Netherlands; 5 Department of Medical Statistics, Leiden University Medical Center, Leiden, the Netherlands; 6 School of Medicine and Biomedical Sciences, The University of Sheffield, Sheffield, UK

## Abstract

**Introduction:**

Progression of joint destruction in rheumatoid arthritis (RA) is partly heritably; 45 to 58% of the variance in joint destruction is estimated to be explained by genetic factors. The binding of RANKL (Receptor Activator for Nuclear Factor κ B Ligand) to RANK results in the activation of TRAF6 (tumor necrosis factor (TNF) receptor associated factor-6), and osteoclast formation ultimately leading to enhanced bone resorption. This bone resorption is inhibited by osteoprotegerin (OPG) which prevents RANKL-RANK interactions. The OPG/RANK/RANKL/TRAF6 pathway plays an important role in bone remodeling. Therefore, we investigated whether genetic variants in *OPG, RANK, RANKL* and *TRAF6* are associated with the rate of joint destruction in RA.

**Methods:**

1,418 patients with 4,885 X-rays of hands and feet derived from four independent data-sets were studied. In each data-set the relative increase of the progression rate per year in the presence of a genotype was assessed. First, explorative analyses were performed on 600 RA-patients from Leiden. 109 SNPs, tagging *OPG, RANK, RANKL* and *TRAF6,* were tested. Single nucleotide polymorphisms (SNPs) significantly associated in phase-1 were genotyped in data-sets from Groningen (Netherlands), Sheffield (United Kingdom) and Lund (Switzerland). Data were summarized in an inverse weighted variance meta-analysis. Bonferonni correction for multiple testing was applied.

**Results:**

We found that 33 SNPs were significantly associated with the rate of joint destruction in phase-1. In phase-2, six SNPs in *OPG* and four SNPs in *RANK* were associated with progression of joint destruction with *P*-value <0.05. In the meta-analyses of all four data-sets, RA-patients with the minor allele of *OPG*-rs1485305 expressed higher rates of joint destruction compared to patients without these risk variants (*P* = 2.35x10^−4^). This variant was also significant after Bonferroni correction.

**Conclusions:**

These results indicate that a genetic variant in *OPG* is associated with a more severe rate of joint destruction in RA.

## Introduction

Rheumatoid Arthritis (RA) is an autoimmune disorder that affects 0.5-1% of the population and is associated with significant morbidity, disability and costs for society. Radiographic joint destruction reflects the cumulative burden of inflammation and is conceived as an objective measure of RA severity [1]. The degree of joint destruction varies significantly between patients. The processes behind this difference are incompletely understood. Inflammatory markers and auto-antibodies are known risk factors for joint destruction but explain approximately 30% of the total variance in joint destruction [2]. A twin study suggested that genetic factors influence the severity of joint destruction in RA and a recent study in the Icelandic RA-population estimated the heritability of the rate of joint destruction around 45-58% [3,4]. Hence, to increase the understanding of progression mediating disease processes, it seems valuable to study genetic variants that could predispose to joint destruction in RA.

**Figure 1:**
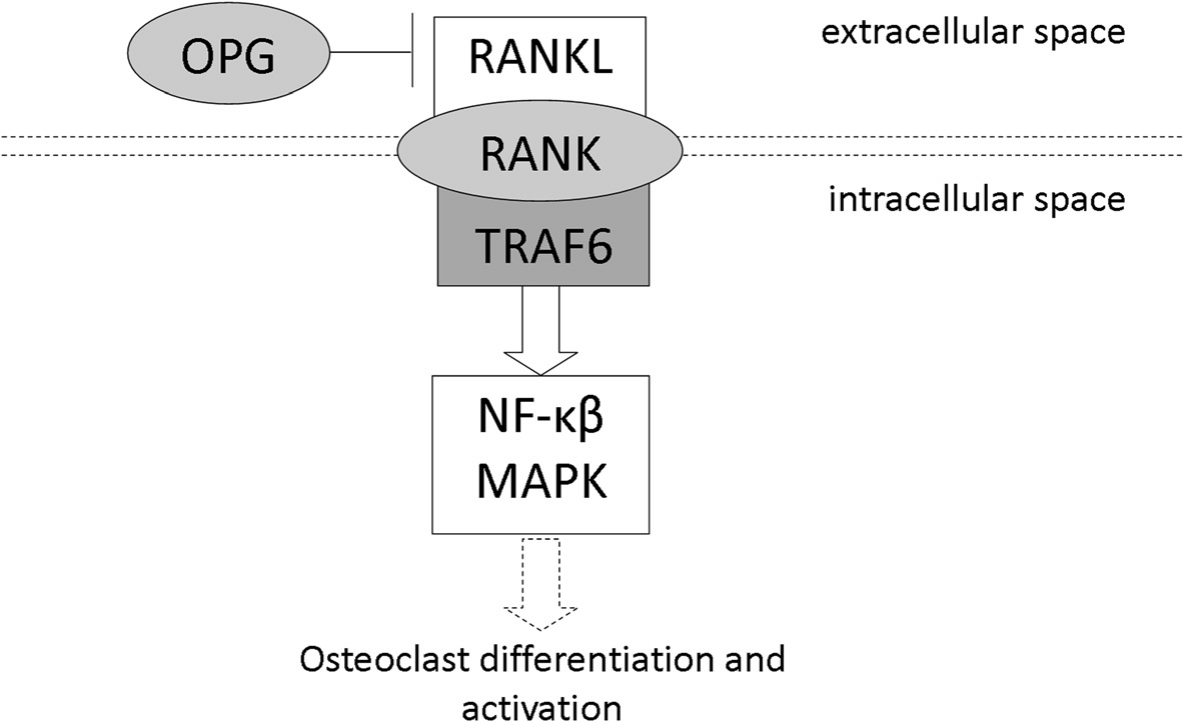
**Schematic presentation of the OPG/RANK/RANKL/TRAF6 pathway in osteoclasts.** The RANK signaling cascade is initiated upon the binding of RANKL to the extracellular domain of RANK which panes the signal along to TRAF6. The activation of TRAF6 initiates pathways leading to the activation of several transcription factors (among which NFκβ and MAP kinase mediators), which contribute to osteoclast differentiation, activation and survival. OPG is able to prevent the interaction between RANKL and RANK.

The balance between osteoblast and osteoclast activity is crucial for healthy bone and is disturbed in systemic or local conditions that affect the skeleton such as osteoporosis or RA. Figure 1 schematically depicts the OPG/RANK/RANKL/TRAF6 pathway which mediates osteoclast related bone loss. RANKL (Receptor Activator for Nuclear Factor κ B Ligand) is expressed and released by osteoblasts and activated T lymphocytes [5]. RANKL promotes osteoclast formation and perpetuate their function and survival through binding of RANK (Receptor Activator of Nuclear Factor κ B). Subsequently, the signal of RANK is mediated by TRAF6, a member of the TNF receptor associated factor (TRAF) protein family, which functions as a signal transducer in the NF κ β family [6]. The process of osteoclast formation and bone resorption is also regulated by OPG (osteoprotegerin), which is secreted by osteoblasts. By binding of OPG to RANKL, activation of the RANK receptor is inhibited.

The net bone loss in RA suggests that there is an imbalance in the OPG-RANKL axis favoring bone resorption and resulting in erosions [6-8]. Recent studies showed that in RA RANKL is, amongst others, expressed in cultured synovial fibroblasts, chondrocytes and by CD4+ and CD8+ T lymphocytes [5,9-11]. In addition, the ratio of OPG/RANKL serum levels is associated with joint destruction in RA [12]. Furthermore, several studies have observed an association of genetic variants in *OPG, RANK* or *RANKL* with bone mineral density and osteoporosis [13-16]. Together, these data led us to hypothesize that genetic variants in *OPG, RANK, RANKL* and *TRAF6* are associated with the severity of joint destruction in RA. We tested this hypothesis using four data-sets of European RA-patients with longitudinal radiological data on joint destruction. All data-sets included patients that were diagnosed in a period when treatment strategies were less aggressive and disease activity was less controlled than today. These conservative treatment strategies made these data-sets suitable for the present study as the natural course of disease was less inhibited.

## Methods

### Study population

Four data-sets consisting of adult European RA-patients were studied. RA was defined according to the 1987 ACR criteria in all data-sets except for the Lund data-set where the 1958 ACR-criteria were used. X-rays of both hands and feet were available for all patients (Table 1). All patients gave their informed consent and approval was obtained from the local Ethical Committee of each hospital (METC Leiden, EPN Lund, METC Groningen, COREC Sheffield).

**Table 1:** Characteristics for each data-set

**Cohort**	**Leiden-EAC**	**Groningen**	**Sheffield**	**Lund**
	**(n = 600)**	**(n = 275)**	**(n = 391)**	**(n = 147)**
**Year of diagnosis**	1993-2006	1945-2001	1938-2003	1985-1990
Follow-up years*	7 years	14 years	Not applicable*	5 years
**Total no. of X-ray sets**	2,846	862	391	781
**Method of scoring**	SHS	SHS	Larsen	Larsen
**Female n (%)**	412 (69)	194 (71)	290 (73)	98 (67)
**Age at diagnosis, mean ± SD**	56 ± 16	49 ± 13	46 ± 13	51 ± 12
**ACPA + n (%)**	323 (55)	160 (80)	302 (79)	114 (80)
**Rheumatoid factor n (%)**	343 (59)	258 (94)	N/A*	115 (81)

*SHS* Sharp-van der Heijde score.

*Data of Leiden-EAC, Groningen and Lund were from baseline onwards during respectively 7, 14 and 5 years of follow-up. The data of Sheffield were collected once during the disease period, the mean disease duration was 15 years (range 3–65 years).

N/A not available.

#### Leiden-early arthritis clinic cohort (Leiden-EAC)

This cohort contained 600 early RA-patients from the western part of the Netherlands, who were included between 1993 and 2006 [2]. Arthritis patients were included at the first visit at the outpatient clinic and yearly followed. Blood samples were collected at baseline. DNA was extracted en preserved for later usage. X-rays were taken at baseline and on yearly follow-up visits during 7-years. In total, 2,846 sets of hands and feet X-rays were available. All X-rays were chronologically scored by one experienced reader who was unaware of genetic or clinical data using the Sharp-van der Heijde scoring method (SHS) on hands and feet [17]. This method quantifies both joint-space-narrowing, a feature of cartilage loss, and bony erosions. It is a semi-quantitative method, the maximal total scores is 448. 499 randomly selected X-rays were scored twice. The correlation coefficient (ICC) within the reader was 0.91. The treatment of these patients could be divided into three treatment periods. Patients included in 1993–1995 were initially treated with NSAIDs, patients included in 1996–1998 were initially treated with chloroquine or sulphasalazine and patients included after 1999 were promptly treated with methotrexate or sulphasalazine.

#### Groningen

The second set of data involved 275 RA-patients from the Northern part of the Netherlands that were diagnosed in 1945–2001. The follow-up duration after diagnosis was limited to 14-years. The mean number of X-ray sets (hands and feet) per patient was 3.1 (with a maximum of eight X-rays per patient). The total number of sets of X-rays was 862. The X-rays were scored chronologically by one of two readers using SHS. ICCs within readers were >0.90 and between readers 0.96. The development of joint destruction was significantly different for patients included after 1990 compared to patients included before 1990. This observation is in line with the introduction of early initiation of treatment with Disease Modifying Anti-Rheumatic Drugs (DMARDs) after 1990.

#### Sheffield

The third set of patients concerned 391 RA-patients from the area of Sheffield, UK. RA-patients with X-rays available were recruited from the Rheumatology department of the Royal Hallamshire Hospital in Sheffield between 1999 and 2006 [17]. RA-patients were assessed once during their disease course. The mean (±SD) disease duration at assessment was 15 ± 11 years (range 3–65 years). X-rays of hands and feet were scored by one reader using a modification to Larsen’s score [18]. This method quantifies the severity of bony erosions and joint space narrowing in one score, both elements are not scored separately. Ten percent of films were scored twice to quantify the intra-observer variation by a weighted kappa score which was 0.83 [19].

#### Lund

This cohort concerned 183 Swedish early RA-patients that were prospectively followed yearly during 5-years, of which 147 had X-rays and DNA available [18,20]. Patients were recruited from primary care units in the area of Lund during 1985–1989. X-rays of hands and feet were taken at study start and annually for 5-years, resulting in a total of 781 sets of X-rays. X-rays were scored chronologically according to Larsen by one of two readers [21]. The ICC between the readers determined on 105 X-rays was 0.94. In the inclusion period, immediate DMARD-therapy was not common and at 5-years follow-up still a substantial proportion of the patients were not treated with a DMARD. The most commonly used DMARDs were chloroquine, D-penicillamin, sodium aurothiomalate and auranofin [22].

### SNP selection and genotyping

The region of *OPG, RANK, RANKL* and *TRAF6* plus the haplotype blocks up- and downstream these genes were tagged by the algorithm of HaploView [23]. One SNP in *OPG*, two in *RANK* and one in *TRAF6* were known to be amino acid changing SNPs; respectively rs2073618, rs1805034, rs8092336 and rs3740958. Eight SNPs were associated with bone mineral density in the hip or spine in previous studies; *OPG* rs6993813 [13], rs646980 [13], rs4355801 [14] and rs2073618 [15]; *RANK* rs3018362 [13] and rs884205 [16]; *RANKL* rs9594738 [13] and rs9594759 [13]. All these SNPs were forced to include. Pairwise tagging SNPs were selected from the CEPH/CEU hapmap data-set (phase II, release 21, NCBI build 35) using haploview software (MAF >0.05, pairwise r2 > 0.8). In total 109 SNPs captured *OPG* (34)*, RANK* (54), *RANKL* (21) and *TRAF6* (17). Multiplex SNPs arrays were designed using Illumina Golden Gate platform, according to the protocols recommended by the manufacturer [Illumina, San Diego, CA]. Three SNPs could not be designed; rs10505348, rs7239667 and rs9951012. Proxies were sought and found for rs10505348:rs4355801 (r^2^ = 0.80), for the other two SNPs no good proxy existed.

Software supplied by Illumina was used to automatically identify the genotypes. Each 96-wells plate consisted of 1 positive and 1 negative control, which were all indeed tested positive and negative. Clusters were evaluated and all doubtful calls were checked; after manually evaluating the spectra of each cluster, the genotypes were accepted, recalled or rejected. At least 12% of the genotypes were assessed in duplicate, with an error rate of <2.5% for all SNPs. Genetic sex was checked with the reported sex in the database. SNPs were selected if the success rate were ≥95% and the Hardy-Weinberg equilibrium (HwE) p-value > 0.001.

SNPs that had a clear and significant association with joint destruction in the first cohort were selected to be genotyped in the other three data-sets. The SNPs were genotyped as a part of multiplex SNPs arrays designed with Sequenom iPLEX, according to the protocols recommended by the manufacturer [Sequenom, San Diego, California]. Software supplied by the same manufacturer was used to automatically identify the genotypes. Two SNPs could not be designed but full proxies (r^2^ = 1.0) were typed instead (rs17666267:rs9959310 and rs1564861:rs3134057). Each iPLEX consisted of at least 9 positive and 9 negative controls, which were indeed tested positive and negative. All doubtful calls were checked manually, DNA samples with >30% failed SNPs were excluded from analysis (n = 31). At least 5% of the genotypes were assessed in duplicate, with an error rate of <1%. SNPs were selected if the success rate were ≥95% and HwE p-value > 0.001.

### Statistical analysis

**Figure 2:**
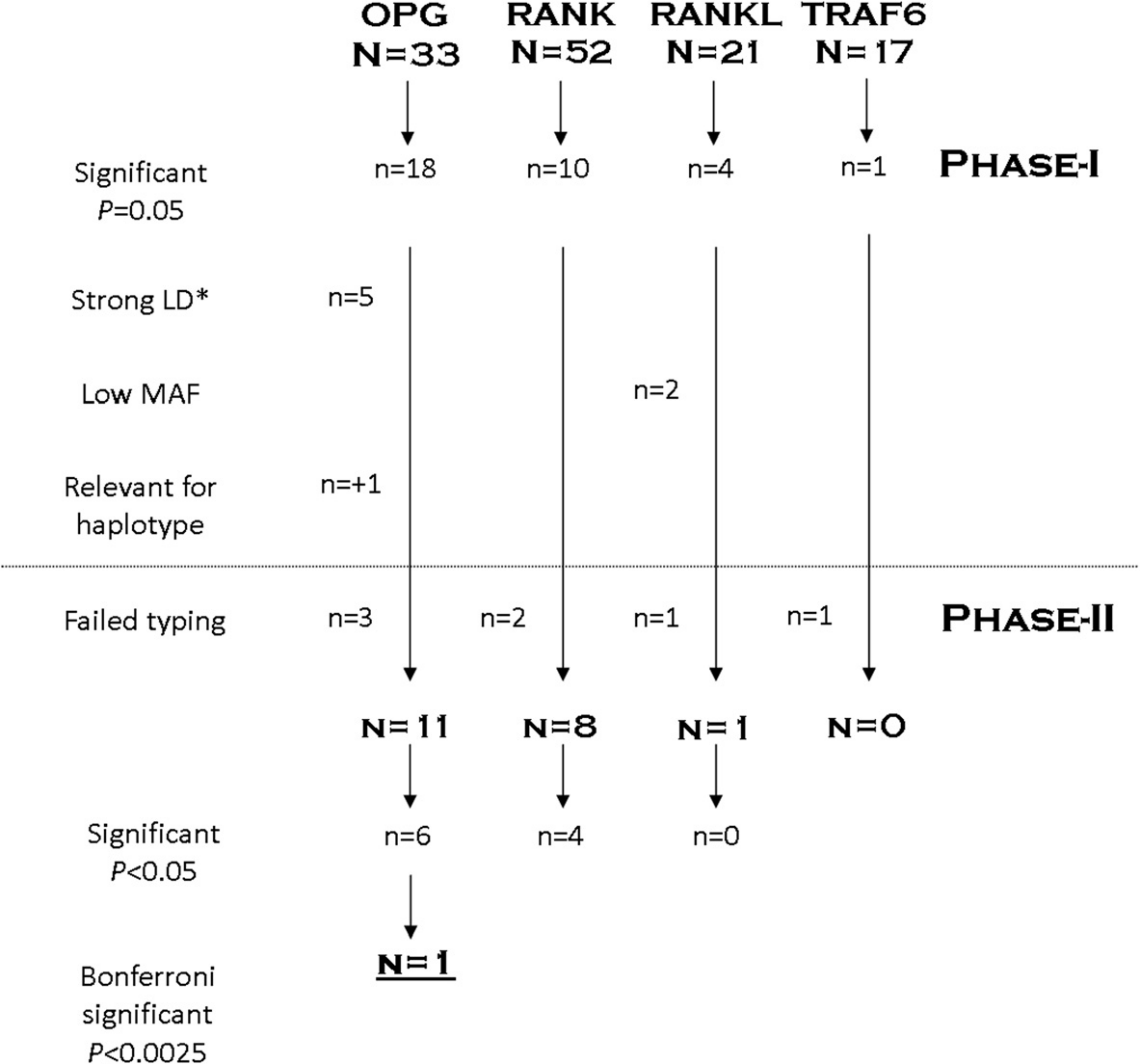
**Schematic depiction of the SNPs selection process.** *LD is linkage disequilibrium, some SNPs were in strong correlation in the dataset of phase-1 as calculated with R^2^ in haploview, or were part of a haplotype were other SNPs caused the significant association.

Associations between genotypes and radiographic joint destruction were analyzed. Two phases were carried out. An overview of the SNP selection process is provided in Figure 2. First, an explorative analysis was performed in the Leiden-EAC. In this data-set the tagged SNPs were tested in two ways; additively and recessively. Since phase-1 was an explorative phase no correction for multiple testing was applied yet and SNPs with a p-value <0.05 were studied in phase-2.

Significant SNPs from phase-1 were preceded in phase-2 if they had a high enough minor allele frequency and if the effect was independent of other SNPs (see Additional file 1: Table S1). In phase-2 SNPs were analyzed in three independent data-sets and meta-analyses of all four data-sets. In the present study the power to detect genetic effects is a function of the number of patients and the number of measurements per patient studied [24,25]. All three data-sets studied to verify the results of phase-1 contained (individually and combined) less X-rays than the initial data-set. Consequently, the power to replicate findings in each data-set individually as well as in the three replication data-sets together was expected to be limited due to a lower number of X-rays than in the discovery data-set. Therefore it was decided to test the SNPs in each data-set separately taking advantage of the specific data-set characteristics and to subsequently perform a meta-analysis on the results to determine the association of the SNPs with the rate of joint destruction. An inverse variance weighting meta-analysis testing for fixed effect [26,27] was performed in Stata, version 10.1. In phase-2 analyses were performed either additively or recessively depending of the findings of phase-1. To provide insight into the heterogeneity of the cohorts the *I*^2^ for heterogeneity was calculated.The choice for a fixed effect model was guided by the argument of Lebrec et al. [27] that the heterogeneity is of less importance in genetic studies when the search for a significant association in the first studies is of more importance than the magnitude of the effect size. Also large GWAs on RA susceptibility had used fixed effect models [28]. In subanalyses a random effect model was also used.

For the analyses in Leiden-EAC, Groningen and Lund a multivariate normal regression model for longitudinal data was used with radiological score as response variable (see detailed descriptions elsewhere [29]). Adjustment variables were entered based on their association with joint destruction; in Leiden-EAC age, gender and the described treatment periods, in Groningen age and inclusion ≤/>1990, as proxy for DMARD-therapy and in Lund adjustments were made for age.

In the Sheffield data-set, each patient had a set of hands and feet X-rays at one time-point. To make the scores comparable to the other data-sets, the estimated yearly progression rate was calculated, by dividing the total Larsen by the number of disease years at time of X-ray [30]. The SNP association was tested in a linear regression analysis with log-transformed estimated yearly progression rate as outcome variable. No adjustments were applied as none of the tested variables was significantly associated with joint destruction.

In all data-sets, the radiological scores were log-transformed to obtain a normal distribution. Since the analyses were performed on the log-scale, the resulting coefficient on the original scale indicates how many fold the joint destruction increased per year of follow-up. Over a follow-up period of n years the coefficient increases to the power of n.

Testing multiple SNPs on one data-sets leads to inflation of the p-value. It is debatable which multiple testing correction is best to use. In the current study the most conservative method was applied, the Bonferroni method, to reduce the chance on false-positive findings as much as possible. Since phase-1 was used as identification phase, this correction was applied to the number of variants tested in phase-2.

### Haplotype analyses

Haplotypes in *OPG, RANK, RANKL* and *TRAF6* were studied. Haplotype blocks for the tag-SNPs were defined with Gabriel’s method [31]. Haplotypes were assigned to each individual using PLINK 1.06 requiring a probability >0.8. Analyses of the haplotypes were performed with methods similar to those used for the analyses of the individual SNPs by now testing the presence of a haplotype compared to the absence of the haplotype.

## Results

### Phase-1; SNP identification

**Figure 3:**
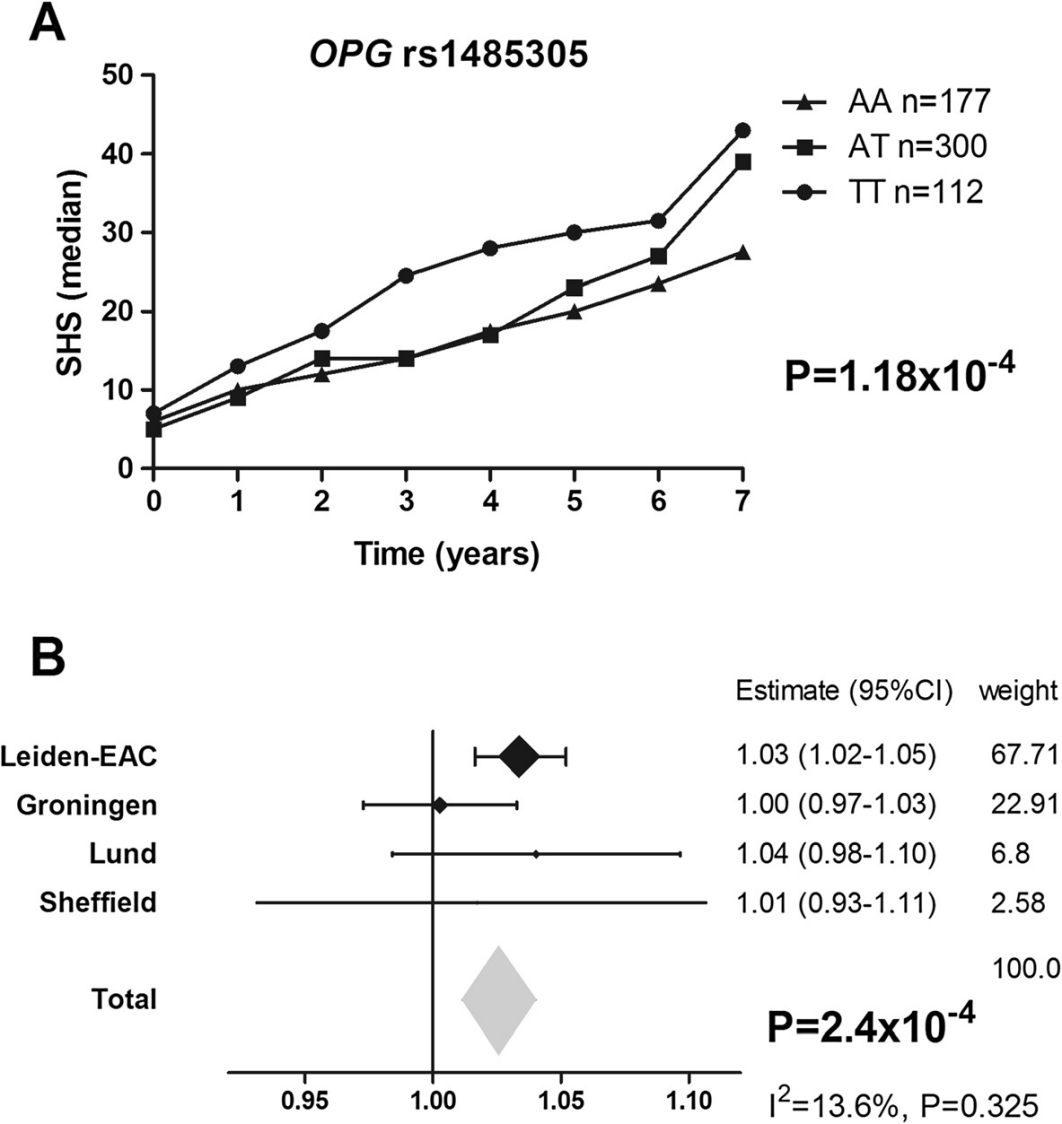
**Depicted is *OPG-*rs1485305 in the Leiden-EAC (A) and in the meta-analysis on all data-sets (B).** SHS = Sharp-van der Heijde score. The effect sizes are the estimated relative progression rates per year for the presence of the minor allele for *OPG* compared to patients without the minor allele. **A)** The presence of the minor variant of *OPG*-1485305 (T) is associated with a 1.03 fold rate of joint destruction per year compared to patients with the common genotypes in the Leiden-EAC. Since the effect sizes increases by the power of the number of follow-up years, these patients have a 1.23 (1.03^7) fold rate of joint destruction over 7-years, in other words a 23% higher rate of joint destruction. **B)** The meta-analysis is based on a fixed effect model, which is applied to genetic studies to test whether there is statistically significant effect; generalizability of the effect is of less importance. Consequently, this method is less suitable to estimate the effect size overall. Therefore, the estimated effect of the meta-analysis is depicted in gray. The *I*^*2*^ was 13.6% and the p-value for heterogeneity was 0.325. The p-value for a random model was 0.004 and the effect size 1.23 (see also Additional file 3: Table S3).

123 tagging SNPs in *OPG* (n = 33)*, RANK* (n = 52)*, RANKL* (n = 21) and *TRAF6* (n = 17) were genotyped. Eleven SNPs were not analyzed because of a low typing success rates and three were out of HwE. From the 109 analyzed SNPs, 33 SNPs were significantly associated with joint destruction (see Additional file 1: Table S1); eighteen SNPs were located in *OPG*, ten in *RANK*, four in *RANKL* and one in *TRAF6*. The associations of *OPG* SNPs were most prominent in the additive analyses. For *RANK, RANKL* and *TRAF6* mainly recessive associations were observed (see Additional file 1: Table S1). The effect sizes observed represent the estimated relative progression rates per year. Consequently over a follow-up of a certain number of years, the effect sizes increases by the power of the number of follow-up years. For example, the estimate of 1.03 fold rate of joint destruction per year of the minor variant of *OPG*-1485305 (T) compared to patients with the common genotypes equals 1.23 (1.03^7) fold rate of joint destruction over 7-years. In other words patients carrying one minor allele had over 7-years a 23% higher rate of joint destruction (Figure 3).

Haplotype analyses were performed in addition to SNPs analyses. In phase-1, four haplotypes from one haplotype block in OPG were identified as possible more informative than the individual SNPs located in these haplotypes (see Additional file 2: Table S2) To tag these haplotypes in phase-2, one non-significant SNP of phase-1 was also typed in phase-2, rs1905785.

### Phase-2; meta-analysis

Of the 33 significant SNPs from phase-1 19 were subsequently typed and analyzed in phase-2 since they had a high enough minor allele frequency and their associations were independent of other SNPs (see Additional file 1: Table S1 and Additional file 3: Table S3 for detailed information). Therefore these nineteen SNPs plus one SNP to allow haplotype analysis were studied in phase-2. As expected due to insufficient power of the replication cohorts, the 95% confidence intervals in each of the three cohorts separately all included 1 (see Additional file 3: Table S3). The SNPs were subsequently analyzed in all 1,418 patients in an inverse variance weighting meta-analysis. Here, ten SNPs (six located in *OPG* and four in *RANK*) were significantly associated with rate of joint destruction (0.04 > *P* > 2.4×10^−4^). After correction for testing 20 SNPs using the Bonferroni method, one SNP was significantly associated with the rate of joint destruction; *OPG*-r1485305 (Figure 3). Patients carrying at least one minor allele of *OPG*-rs1485305 (T) had a higher rate of joint destruction as compared to patients without this minor allele (*P* = 2.35×10^−4^,uncorrected *P*-value).

When the association of *OPG*-rs1485305 with the rate of joint destruction was studied in ACPA-negative and ACPA-positive patients separately in the Leiden-EAC, rs1485305 was significantly associated with progression of joint destruction in ACPA-negative patients (1.29 95% CI 1.10-1.50, *P* = 0.001) but not in ACPA-positive patients although a similar trend was observed (1.14 95% CI 0.97-1.34, *P* = 0.11). Also when the analysis in the total group of RA –patients was adjusted for ACPA and rheumatoid factor the association remained significant (1.20 95% CI 1.07-1.35, *P* = 0.02). Similarly, adjusting for the level of inflammation measured by C-reactive protein levels at baseline) did not affect the effect size and significance was maintained (1.29 95% CI 1.14-1.46, *P* = 0.0005). This suggests that the association between *OPG*-rs1485305 and radiological progression in the Leiden data was not confounded by auto-antibodies or CRP-levels. Unfortunately, our data on these adjustment factors were too limited to confirm this with the other cohorts.

In phase-2, none of the tested haplotypes provided additional information to the results of the individual SNPs (see Additional file 2: Table S2).

## Discussion and conclusion

The variance in joint destruction between RA-patients is considerable and the mechanisms driving these differences are thus far scarcely understood. Part of the severity of joint damage is explained by the cumulative levels of inflammation [32] though this explanation is incomplete. We reasoned that individual susceptibility of bones to erode may affect the severity of joint destruction in RA as well. We therefore studied the association of genetic variants in *OPG, RANK, RANKL* and *TRAF6* with joint destruction as the products of these genes together constitute a pathway that is crucial in osteoclastogenesis and bone resorption. One SNP, *OPG*-rs14085305, was observed to significantly associate with progression of joint destruction in RA.

The association between *OPG*-rs14085305 with progression of joint destruction in RA was independent of auto-antibody positivity. However, after stratification for ACPA the association was only significant in the ACPA-negative group. This seemingly counterintuitive result could be explained by a strong effect of the association in the ACPA-negative group, by which the multivariable analysis remained significant. Furthermore, since the effect sizes iwere quite similar in the ACPA-positive and ACPA-negative subgroups, it is also possible that the association in ACPA-positive patients was insufficiently powered to obtain statistical significance in this subgroup. Larger datasets of ACPA + RA-patients would be required to further elucidate the association between rs14085305 and joint damage progression in ACPA + RA.

OPG is expressed in several cells and tissues among which osteoblasts, chondrocytes and bone marrow. Serum OPG is decreased in the synovium and serum of RA-patients [33]. Low serum OPG/RANKL ratio’s have been associated with progression of joint destruction [12]. In addition, low OPG/RANKL ratio’s in cartilage are associated with the degree of joint destruction is situated in the 5-UTR flanking region of *OPG.* Thus far no functional data on this variant exist, hence the mechanism by which rs1485305 affects OPG expression or function is yet unknown. Nonetheless, *OPG*-rs1485305 was recently also observed to associate with bone mineral density loss, which strengthens the relevance of this SNP in relation to bone and joint disease [34].

Our group recently performed a genome-wide study on joint damage progression in ACPA-positive RA-patients [35] and evaluated the genetic variants included on the Immunochip in relation to joint damage progression in RA [36]. In these studies the current variant OPG-rs1485305 was not identified as a risk factor for joint damage progression as this variant was not (also no proxies with r^2^ > 0.80) included in both these genotyping platforms.

An advantage of the four studied data-sets is that the evaluated RA-patients were treated in an era when treatment was not as aggressive as nowadays. Hence, the radiologic progression rates of the studied patients are more reflective of the natural course of RA than that of recently treated patients. Some data-sets included patients from different periods that had received different treatment regiments potentially affecting an association with progression of joint destruction. Since treatment may be an effect modifier masking associations with radiological progression, the analyses in these data-sets were adjusted for inclusion period as proxy for treatment.

Replication data-sets are ideally larger than the initial data-set, since effects sizes are generally smaller at a replication stage. A limitation of the present study is that we were not able to include replication data-sets that contained more X-rays than the initial data-set and of which the RA-patients were “conventionally” treated. Most likely, few of such longitudinal data-sets exist. In one data-set only one radiograph per patient was available; it is known that this results in less precise estimations of the progression rate compared to having serial radiological measurements [37]. This was here also depicted by a broader confidence interval of the effect estimate of the Sheffield data. Nonetheless the results of a meta-analysis on rs1485305 without the Sheffield data was still significant (p = 0.0024 data not shown).

Since the number of patients and the number X-rays of each data-set separate were insufficient to allow well powered analyses, the data of the different replication cohorts were summarized in inverse variance weighting meta-analyses. Notably, also the replication data-sets combined contained less radiological measurements than in phase-1. Importantly, the effects of *OPG*-rs1485305 went into the same direction in each dataset supporting the validity of the results. In addition, the heterogeneity (*I*^2^*)* of the data for *OPG*-rs1485305 was only 13.6%, making it less likely that *OPG*-rs1485305 is a false positive finding due to differences between the four cohorts. This *I*^2^ supports the use of a fixed effects meta-analysis, but also when a random effect model was used OPG-rs1485305 remained significant (Additional file 3: Table S3).

To further prevent false positive findings due to performing multiple comparisons, data were corrected for multiple testing using the Bonferroni method. This was done in phase-2, since phase-1 was used as discovery phase. However, the association of *OPG*-rs1485305 with the rate of joint destruction would also have remained significant when Bonferroni correction would have been applied in phase-1 correcting for 109 SNPs. This further consolidates the validity on the results on *OPG*-rs1485305.

Interestingly, several other studies, among which genome-wide association studies, have revealed several genetic variants in RANKL/RANK/OPG to associate with bone mineral density or osteoporosis. Therefore during the tagging and SNP selection phase, such variants were forced into the selection. Interestingly (except for rs1485305) none of these variants were significantly associated with progression of joint destruction in phase-2. Conceptually, the balance between osteoblast and osteoclast activity is crucial both in osteoporosis and joint destruction in RA but the individual genetic variants predisposing to such systemic or local bone loss are largely dissimilar.

During the SNP selection phase, three coding SNPs were also prioritized; these three were also not significantly associated after correction for multiple testing in phase-2. A coding variant in *RANK*, rs8092336, was significantly associated with joint destruction in phase-1 and phase-2, but did not remain significant after correction for 20 tests. The same was observed for a coding SNP in *OPG*, rs2073618, which has also been associated with bone mineral density [15]. These two SNPs could potentially be associated with progression of joint destruction, but the chance of a Type-I error is too large to conclude this on the basis of current data.

Finally, *TRAF6* was chosen as candidate gene because it is a relevant signal transducer in the Nuclear Factor κ B pathway. Rs540386 in *TRAF6* was previously also identified as a risk locus for RA susceptibility, though *TRAF6* was not genome-wide significant in a recent study on more than 11,000 cases and 15,000 controls [29,38]. Our candidate gene study had started a year before the first report of *TRAF6* and RA susceptibility was published, hence this report had not affected the choice of *TRAF6* as candidate gene. The susceptibility SNP rs540386 is in close LD (r^2^ = 0.94) with one of the tag SNP evaluated in the current study (rs11033647). This SNP was not associated with the rate of joint destruction in our analyses. Although this could be a false-negative finding, it is also possible that different genetic variants are involved in RA-susceptibility and the progression of joint destruction.

The current study included patients only. Whether the rs1485305 is also relevant for RA-susceptibility was not studied here. Nonetheless, several genome-wide studies have been performed on RA-susceptibility and >40 genetic susceptibility factors have been identified but rs1485305 in OPG was not one of them. Presumably different genetic factors are involved in developing RA and in progression of structural damage.

In conclusion, with a candidate gene approach evaluating patients of four different cohorts, we found association of a genetic variant in *OPG* with an increased rate of joint destruction in RA*.* The present data support the role of OPG in joint destruction in RA by indicating that the risk allele of rs1485305 may affect the homeostasis in bone.

## Abbreviations

DMARDs: Disease modifying anti-rheumatic drugs; EAC: Early Arthritis Clinic; HwE: Hardy-Weinberg equilibrium; ICC: Intra-correlation coefficient; MAF: Minor allele frequency; NF: Nuclear Factor; NSAIDs: Non-steroid anti-inflammatory drugs; OPG: Osteoprotegerin; RA: Rheumatoid Arthritis; RANK: Receptor Activator for Nuclear Factor κ B; RANKL: Receptor Activator for Nuclear Factor κ B Ligand; SD: Standard deviation; SHS: Sharp-van der Heijde; SNP: Single nucleotide polymorphism; TRAF6: Tumour necrosis Receptor Associated Factor-6.

## Competing interests

None of the authors has any financial conflicts of interest to declare.

## Authors’ contributions

RK: design, collection, analyses, manuscript (re)writing, final approval. AH: design, collection, manuscript writing, final approval. ND: data collection, analyses, manuscript rewriting, final approval. GW: data collection, manuscript rewriting, final approval. EL: data collection, manuscript rewriting final approval. ML: data collection, manuscript rewriting, final approval. BK: data collection, manuscript rewriting, final approval. RT: data collection, manuscript rewriting, final approval. TS: data collection, manuscript rewriting, final approval. TH: data collection, manuscript rewriting, final approval. EB: data collection, manuscript rewriting, final approval. DR analyses, manuscript rewriting, final approval. JS: analyses, data collection, manuscript rewriting, final approval. JH data analysis, manuscript rewriting, conception final approval. All authors read and approved the final manuscript.

## Supplementary Material

Additional file 1: Table S1 Results of SNP analysis phase-1 in the Leiden-EAC. Click here for file

Additional file 2: Table S2 Haplotype analyses. Click here for file

Additional file 3: Table S3 Results of the significant SNPs of the phase-I, phase-II and the meta-analysis (fixed as well as random effects). The fixed effect model was our main model of choice, guided by the argument of Lebrec et al. [27] that the heterogeneity is of less importance in genetic studies when the search for a significant association in the first studies is of more importance than the magnitude of the effect size. To provide insight into the heterogeneity of the cohorts the I2 for heterogeneity was calculated. In this table also the results of a random meta-analysis is provided. The final conclusion that OPG-rs1485305 is significantly associated with rate of joint destruction is irrespective of which model for meta-analysis is used. Click here for file
